# Combined multiplex polymerase chain reaction-based targeted next−generation sequencing and serum 1, 3-β-D-glucan for differential diagnosis of *Pneumocystis* pneumonia *and Pneumocystis* colonization

**DOI:** 10.3389/fcimb.2025.1611391

**Published:** 2025-09-18

**Authors:** Hansheng Wang, Xiao Chen, Xiaofeng Wu, Qizhen Cao, Yi Wu, Fang Wang, Yunyun Wang, Yanhui Zhou, Yijun Tang, Tao Ren, Meifang Wang

**Affiliations:** ^1^ Department of Pulmonary and Critical Care Medicine, Shiyan Key Laboratory of Major Chronic Respiratory Disease, Hubei Provincial Clinical Research Center for Umbilical Cord Blood Hematopoietic Stem Cells, Taihe Hospital, Hubei University of Medicine, Shiyan, Hubei, China; ^2^ Department of Laboratory, Shiyan Maternal and Child Health Hospital, Hubei University of Medicine, Shiyan, Hubei, China; ^3^ Department of Radiology, Taihe Hospital, Hubei University of Medicine, Shiyan, Hubei, China; ^4^ Department of Cardiothoracic surgery, Taihe Hospital, Hubei University of Medicine, Shiyan, Hubei, China

**Keywords:** *Pneumocystis jirovecii* pneumonia (PJP), multiplex PCR-based targeted next generation sequencing (mp-tNGS), bronchoalveolar lavage fluid (BALF), 1, 3-β-D-glucan (BDG), colonization

## Abstract

**Background and Objective:**

*Pneumocystis jirovecii* pneumonia (PjP) remains an important cause of morbimortality worldwide, and differentiating *Pneumocystis jirovecii* (*P. jirovecii*) infection from *P. jirovecii* colonization (PjC) is crucial for guiding treatment strategies. Multiplex polymerase chain reaction-based targeted next-generation sequencing (mp-tNGS) is a promising tool for identifying lower respiratory tract infections, with a detectable pathogen spectrum that covers more than 95% of clinical infectious cases. This study evaluated mp-tNGS for *P. jirovecii* identification in bronchoalveolar lavage fluid (BALF) samples combined with serum 1,3-β-D-glucan (BDG) level detection to differentiate PjP and PjC.

**Methods:**

A total of 73 patients were enrolled and the final diagnosis was used as a reference criterion, and patients were divided into the PjP group and PjC group. The clinical data and detection performance of mp-tNGS/serum BDG were analyzed.

**Results:**

The median fungal reads (normalized sequence counts) detected by mp-tNGS were 1522.00 (interquartile range [IQR], 581.5, 4898.0) in the PjP group versus 117.00 (IQR, 79.00, 257.00) in the PjC group (*p <*0.0001). Correspondingly, BDG levels were 122.5 (88.75,239.3) pg/ml in PjP patients compared to 59.00 (51.0,79.0) pg/ml in PjC patients (*p <*0.0001). Area under the receiver operator characteristic curve (AUROC) for discriminating PjP from colonization was 0.935 (95% CI: 0.88–0.99) for BALF mp-tNGS and 0.822 (95% CI: 0.72–0.93) for serum BDG. The optimal diagnostic thresholds were determined to be 355 reads for mp-tNGS (sensitivity: 89.1%; specificity: 85.2%) and 84.5 pg/ml for BDG (sensitivity: 85.2%; specificity: 80.4%).

**Conclusion:**

BALF mp-tNGS and serum BDG serve as valuable adjunct diagnostic tools, providing reliable differentiation between *P. jirovecii* colonization and active infection.

## 1 Introduction

*Pneumocystis jirovecii* pneumonia (PjP) is an acute and potentially fatal infection caused by the opportunistic fungal pathogen *pneumocystis jirovecii*, which predominantly affects immunocompromised individuals globally (Grønseth et al., 2021), with a particularly high prevalence among people living with HIV (PLWH) (Thomas and Limper, 2004). The incidence of PjP in PLWH has gradually decreased due to highly effective antiretroviral therapy and prophylactic treatment against *P. jirovecii* (Crothers et al., 2011; Salzer et al., 2018). However, with the extensive use of glucocorticoids, immunosuppressants, radiotherapy and chemotherapy for tumors and organ transplantation, the incidence of PjP has risen significantly in non-HIV-infected populations (Bienvenu et al., 2016; Dunbar et al., 2020). Notably, non-HIV-infected immunocompromised individuals with PjP often exhibit a poor prognosis, characterized by severe clinical manifestations, an acute disease course, and rapid progression (Kanj et al., 2021). It has been reported that the mortality rate of PjP ranges from 10% to 20% in PLWH, whereas it significantly increases to 30%–60% in non-HIV-infected individuals (Morris and Norris, 2012; Stern et al., 2014; Kotani et al., 2017).

Traditionally, in the presence of clinical suspicion, PjP is diagnosed by the microscopic presence of *pneumocysts* in respiratory specimens by the use of staining methods, such as Giemsa, Gomori methenamine silver (GMS), calcofluor white, toluidine blue, and/or immunofluorescence antibody assay (Calderón et al., 2010). However, these methods are limited by suboptimal sensitivity and specificity, as well as prolonged turnaround times (TAT).

BDG, a highly conserved polysaccharide component of fungal cell walls, is synthesized during the biogenesis of the fungal cell wall in numerous pathogenic species. This immunogenic molecule has been established as a reliable biomarker for fungal infections, particularly pulmonary aspergillosis and invasive candidiasis (Obayashi et al., 1995). Although the assay lacks specificity for *Pneumocystis jirovecii*, quantitative detection of serum BDG levels has been established as an adjunctive tool for the diagnosis of PjP (Tasaka et al., 2007; Watanabe et al., 2009; Matsumura et al., 2012; Onishi et al., 2012). Previous studies have established varying diagnostic thresholds for serum BDG in *Pneumocystis jirovecii* detection. Tasaka et al (Tasaka et al., 2007). determined an optimal cut-off value of 31.1 pg/mL, demonstrating a negative predictive value of 0.980 and positive predictive value of 0.610. Notably, Matsumura et al (Matsumura et al., 2012). achieved a better area under the receiver operator characteristic curve (AUROC) of 0.91 and a lower threshold of 15.6 pg/mL, maintaining 100% sensitivity while providing 80.0% specificity for definitive PjP diagnosis. Meta-analysis by Del Corpo et al (Del Corpo et al., 2020). revealed that serum BDG assay demonstrated limited diagnostic utility for PjP, with insufficient pooled sensitivity to exclude infection and inadequate specificity to confirm PjP diagnosis, particularly in high-prevalence populations. Consequently, we recommend that a comprehensive diagnostic strategy integrating serum BDG assay with complementary microbiological or molecular diagnostic modalities is essential for establishing reliable PjP diagnosis, especially in high-risk populations.

With the rapid advancements in molecular biology, techniques such as metagenomic next-generation sequencing (mNGS) and multiplex PCR-based targeted next-generation sequencing (mp-tNGS) are now widely employed to directly detect target organisms in respiratory samples (Sun et al., 2024; Yin et al., 2024; Hsu et al., 2025). A literature search conducted by X. Li et al (Li et al., 2023). identified 9 studies encompassing 1,343 patients, demonstrating that the pooled sensitivity and specificity of mNGS for diagnosing PjP were 0.974 (95% CI: 0.953–0.987) and 0.943 (95% CI: 0.926–0.957), respectively. A large-scale prospective study by Yin et al (Yin et al., 2024). demonstrated that tNGS outperformed mNGS in detecting *pneumocystis jirovecii in* BALF samples. Moreover, compared to mp-tNGS, mNGS has the limitations of high costs, long TAT, human gene interference, and deoxyribonucleic acid (DNA) and ribonucleic acid (RNA) must be separately detected (Miller et al., 2019). However, distinguishing colonization from active infection remains a challenge when using mNGS or tNGS, as positive results may be fungal colonization rather than infection; importantly, PjP is a life-threatening infection in the absence of specific treatment, whereas PjC is a less-severe presentation of asymptomatic colonization (Damiani et al., 2013).

However, the diagnostic performance of combining serum BDG assay and BALF mp-tNGS for differentiating between colonization and infection of *pneumocystis jirovecii* remains unexplored in current literature. In this study, we evaluated the diagnostic performance of BALF mp-tNGS combing with serum BDG for differentiating *P. jirovecii* infection between colonization.

## 2 Patients and methods

### 2.1 Participants and study design

Between January 2022 and December 2024, patients presenting with pulmonary infiltrates of undetermined etiology or clinical suspicion of PjP who received BALF mp-tNGS testing during the study period were enrolled at Taihe Hospital. Patients were excluded if the following criteria were met: (1) administered empiric treatment (therapeutic doses) for PjP prior to the mp-tNGS tests, (2) serum BDG assay were performed more than 7 days after mp-tNGS, (3) other pulmonary fungal infections are present, and (4) incomplete medical record. A complete medical records encompassing demographic characteristics, clinical parameters, radiologic and laboratory findings was collected before the end of the study for all enrolled patients, as follows: (1) underlying diseases (solid organ malignancy, hematologic malignancies, HIV/AIDS status, prolonged use of corticosteroids/immunosuppressants and autoimmune disease); (2) radiographic findings (chest X-ray and high-resolution computed tomography); (3) laboratory results (absolute count of leukocytes/lymphocyte, HIV viral load, hypersensitive-C-reactive protein [hs-CRP], erythrocyte sedimentation rate (ESR), interleukin-6 [IL-6], procalcitonin [PCT] and lactate dehydrogenase [LDH]); and (4) clinical outcomes. Two independent senior clinicians who reviewed and evaluated the patients’ immune status, clinical manifestations, laboratory results, radiologic findings, the mp-tNGS report, and the response to anti-*P. jirovecii* treatment to determine the final diagnosis of PjP. The final diagnosis was used as a reference to which the mp-tNGS and serum BDG results were compared. This study was approved by the Ethics Committee of Taihe Hospital and informed written consent was obtained from each patient.

### 2.2 Definitions

**Figure 1:**
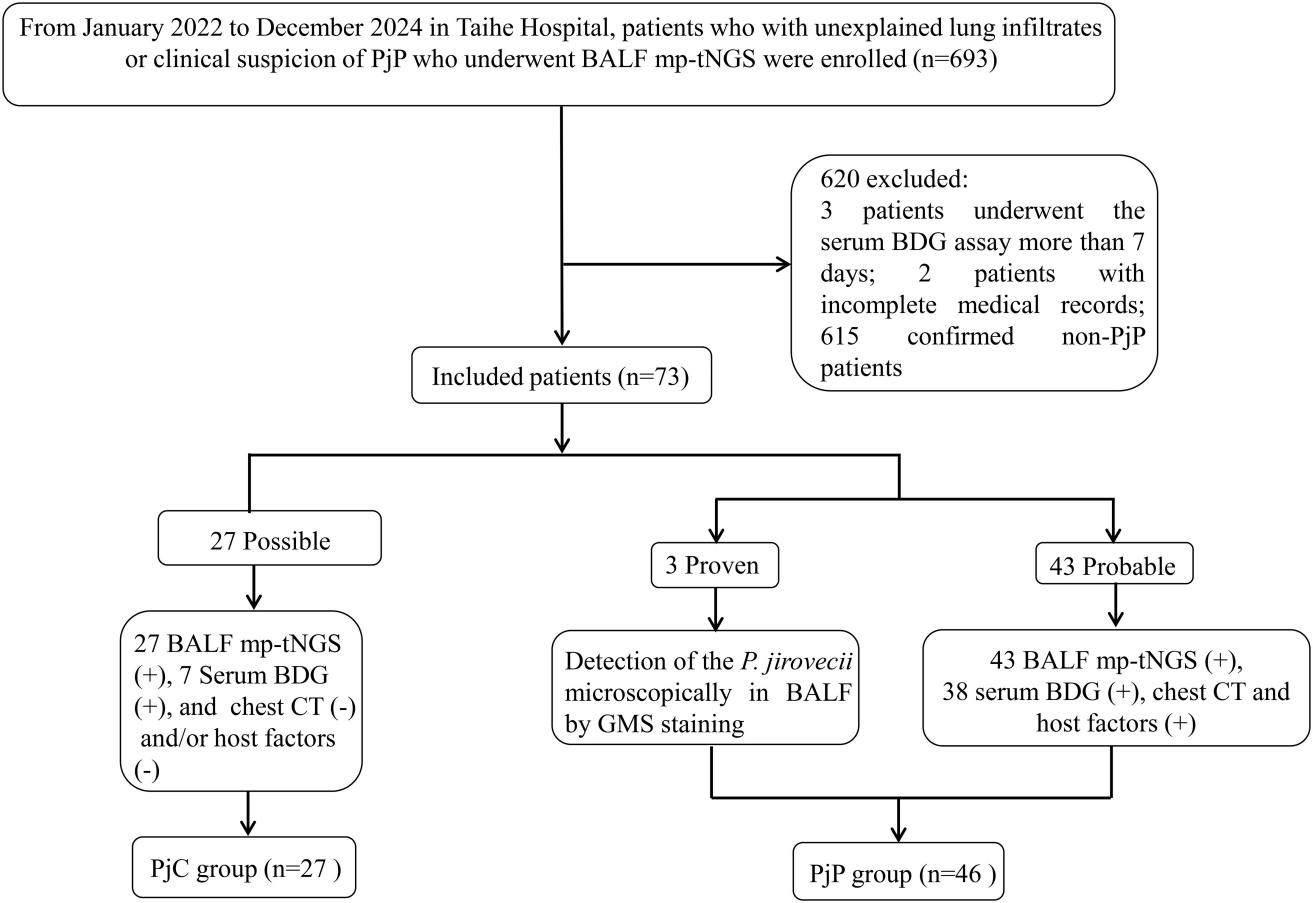
Flowchart for the enrolled and diagnosis of the study population.

PjP classification followed modified EORTC/MSG consensus guidelines for invasive fungal diseases (Donnelly et al., 2020). Cases were categorized as: ***Proven/probable PjP***: BALF demonstrating *P. jirovecii* via microscopy or mp-tNGS, plus clinical symptoms (fever >38°C, dyspnea, dry cough, or room-air SpO_2_ <90%) and characteristic CT findings (bilateral ground-glass opacities/diffuse infiltrates). ***Possible* *PjP:*** BALF mp-tNGS-positive for *P. jirovecii* without corresponding clinical/radiological features. ***Non-PjP:*** Absence of microbiological and mp-tNGS evidence and clinical-radiological suspicion. As shown in **Figure 1**, the Proven and probable PjP cases were grouped into the PjP group; cases with a Possible PjP diagnosis were grouped into the PjC group.

### 2.3 Serum BDG tests

The (1–3)-β-D-glucan detection kit (Dynamiker Biotechnology Co., Ltd., Tianjin, China) was used to assess the levels of serum BDG following the manufacturer’s instructions with a chromogenic method (Yu et al., 2020). A serum BDG level ≥80 pg/ml was considered positive, as per the manufacturer’s recommended cut-off value.

### 2.5 mp-tNGS procedures and bioinformatics analysis

#### 2.5.1 mp-tNGS workflow construction

Mp-tNGS workflow construction, including database integration and primer design, refer to the study of Zhang P et al (Zhang et al., 2024) YIN Y et al (Yin et al., 2024), the mp-tNGS panel covered 198 pathogen targets commonly encountered in clinical scenarios. A full list of the target species identified by the mp-tNGS panel is presented in **Supplementary Table S1**. Based on the integrated database in NCBI, target loci capable of precisely identifying species and strains were selected for primer design. Initially, target genes recommended by classical PCR methods in the literature were chosen, after which conserved and specific regions were assessed by bioinformatics evaluation. The primer design adheres to the following key principles: (1). The GC content (guanine - cytosine content) of primers is set within the range of 40% to 60%. (2). The length of primers is regulated between 18 base pairs and 26 base pairs. (3). The melting temperature (T_m_ value) of primers is designed to be around 60°C. (4). Self-dimers, hairpins, and cross-dimers must be avoided. Primer sets for over 300-plex amplification were designed, and additional primers (≥5) were designed for significant pathogens (e.g., *Mycobacterium tuberculosis complex* [MTBC]) and those that require typing (e.g., SARS-CoV-2). A PCR process was developed and optimized to effectively amplify target signals with high sensitivity.

#### 2.5.2 mp-tNGS nucleic acid extraction

DTT liquefaction reagent (0.1 M) in the same volume as the BALF sample was added to the sampling tube, which was subsequently vortexed, shaken, mixed well, and left for 3–5 min until the sample liquefied. A total of 1.3 mL of liquefied sample was added, 13 μL of exogenous endogenous reagent was added, the mixture was mixed well, the mixture was centrifuged at 12000 rpm for 5min, the supernatant was discarded, 500 μL of the sample was removed, the sample was mixed with a pipette, 500 μL of the sample was added to the bead mill tube of the extraction kit, 50 μL of SDS was added, and the mixture was placed into the sonicator (4700 rpm, oscillated for 45 s, with an intermittent pause of 20 s, and 2 intervals, with a total of 3 oscillations of 135 s) for sonication. The total time of 3 oscillations was 135 s for the wall-breaking treatment. After wall-breaking treatment, the mixture was centrifuged at 12000 rpm for 5min, and 400 μL (manual extraction) or 250 μL (automated extraction) of the supernatant was collected for nucleic acid extraction via the MetaPure DNA & RNA Extraction Kit (KingCreate, Guangzhou, China) and extracted according to the instructions of the kit.

#### 2.5.3 mp-tNGS library construction

The target species covered by the mp-tNGS panel are listed in **Supplementary Table S1**. Library preparation was performed via the RP100™ Respiratory Pathogen Microorganisms Multiplex Testing Kit (KingCreate Biotechnology Co., Ltd., Guangzhou, China). cDNA was synthesized via reverse transcription of the extracted nucleic acids, followed by steps such as target region enrichment, the first round of purification, junction ligation, and the second round of purification to complete library construction. Nuclease-free water (Invitrogen, Waltham, MA, USA) was used as a nontemplate control (NTC) to detect contamination. The generated libraries were quantified via an Equalbit DNA HS Assay Kit (Vazyme Biotech, Nanjing, Jiangsu, China) with an Invitrogen™ Qubit™ 3.0/4.0 (Thermo Fisher Scientific, Waltham, MA, USA) fluorometer to ensure that all the samples had a library density ≥ 0.5 ng/μL or were subjected to library reconstruction. The constructed libraries were pooled according to the assayed concentrations in equal mass. The size of the library fragments was detected by a fully automated nucleic acid protein analyser (Qsep100) via a Standard Cartridge Kit (S2). The operation was carried out according to the instructions of the kit, and the size of the library fragments was approximately 250–350 bp. After library qualification, the pooled library was diluted and denatured according to the kit instructions to prepare a mixed library ready for sequencing, and 500 μL of the mixed library was added to the KM MiniSeqDx-CN Sequencing Kit on the KM MiniSeq Dx-CN Platform (KingCreate, Guangzhou, Guangdong, China).

#### 2.5.4 Bioinformatics analysis

The sequencing data generated were analysed via a bioinformatic workflow. The raw sequencing read data were subjected to a quality control procedure. fastp v0.20.1 (Chen et al., 2018) was employed for adaptor trimming and low-quality filtering with default parameters, and Bowtie2 v2.4.1 (Langmead and Salzberg, 2012) was utilized to map the reads of each sample against the mp-tNGS special authoritatively classified database in very sensitive mode.

### 2.6 Statistical analysis

SPSS software version 26.0 was used for the statistical analysis. The Shapiro-Wilk test was used to assess whether continuous variables followed a normal or non-normal distribution. The continuous variables with normal distribution were expressed as the means ± standard deviations (means ± SDs), and a t test was used for comparison between two groups; non-normally distributed continuous variables were expressed as the median and interquartile range (25th–75th percentiles), and the Mann–Whitney U test was used for comparison between two groups. Receiver operating characteristic (ROC) curves were designed to assess the sensitivity, specificity, PPV (positive predictive value) () and NPV (negative predictive value) for the serum BDG and mp-tNGS. Categorical variables were expressed as n (%) and compared using the chi-square test. Probability values <5% (*p*<0.05) were considered statistically significant. The graphical were generated utilizing GraphPad Prism 9 software (GraphPad, Inc., La Jolla, CA, USA). Optimal cutoffs of 355 reads for mp-tNGS and 84.5 pg/mL for BDG were derived from a mathematical assessment of the ROC curves using an optimization analysis of Youden’s J statistic.

## 3 Results

### 3.1 Demographic and clinical characteristics

During the 35-month study period, A total of 693 patients who underwent bronchoalveolar lavage for BALF sample collection and subsequent mp-tNGS testing were included in the study. Among them, seventy-eight patients with positive *P. jirovecii* mp-tNGS tests were divided into proven/probable PjP group (n=48), possible PjP group (n=30); and negative mp-tNGS in non-PjP group (n=615); serum BDG assay was performed more than 7 days after mp-tNGS in 3 patients, medical records were incomplete in 2 patients. Therefore, a total of 73 cases were ultimately included and classified into the PjP group (n=46) and the PjC group (n=27) based on the final diagnosis (**Figure 1**).

**Figure 2:**
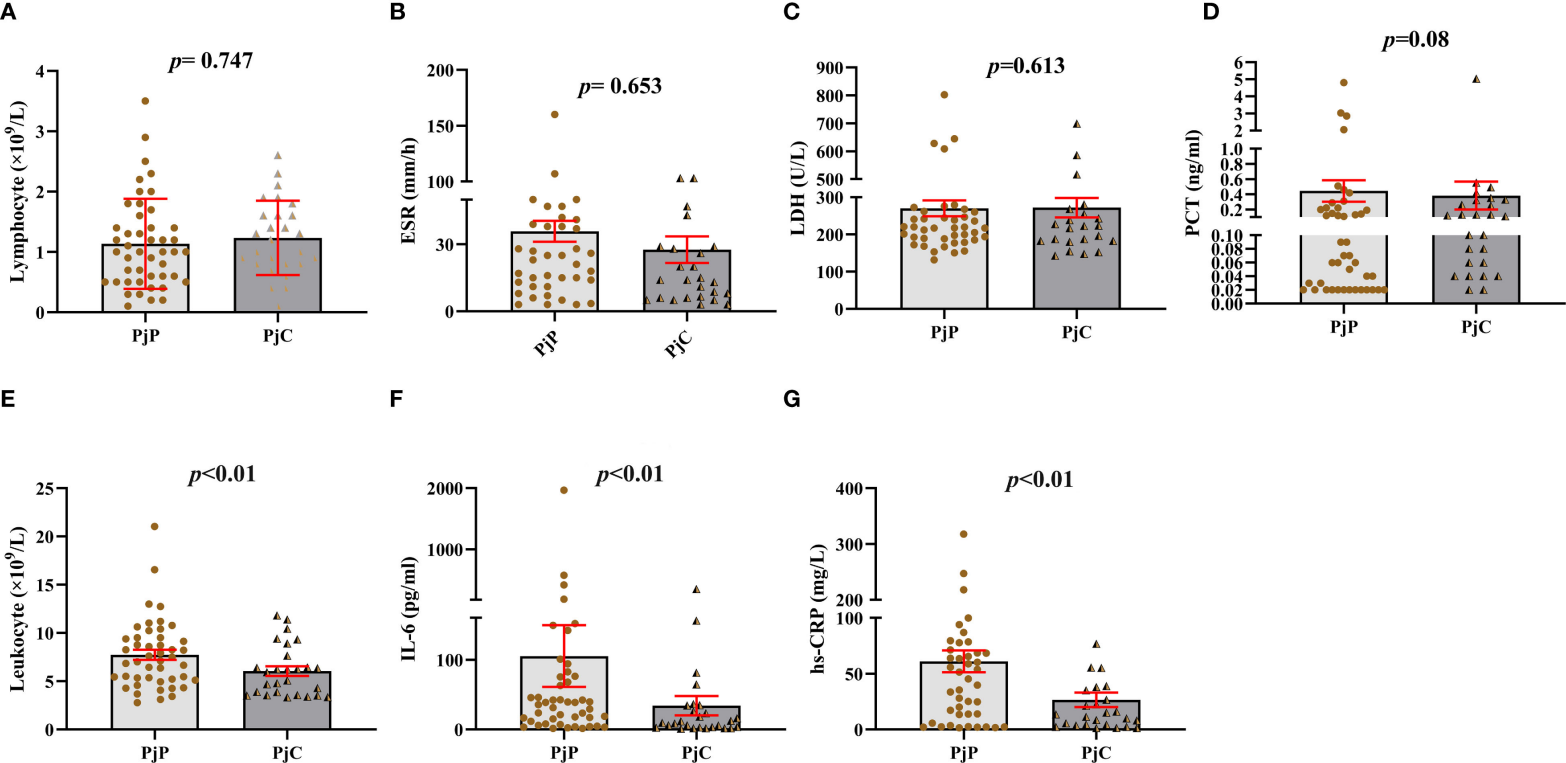
Blood analysis of leukocyte and lymphocyte counts, erythrocyte sedimentation rate (ESR), lactate dehydrogenase (LDH), hypersensitive C-reactive protein (hs-CRP), procalcitonin (PCT), and interleukin-6 (IL-6) levels in *Pneumocystis jirovecii* pneumonia (PjP) and *Pneumocystis jirovecii* colonization (PjC) groups. **(A)** Comparison of the lymphocyte counts between PjP and PjC groups. **(B)** Comparison of the ESR levels between PjP and PjC groups. **(C)** Comparison of the LDH levels between PjP and PjC groups. **(D)** Comparison of the PCT levels between PjP and PjC groups. **(E)** Comparison of the leukocyte counts between PjP and PjC groups. **(F)** Comparison of the IL-6 levels between PjP and PjC groups. **(G)** Comparison of the hs-CRP levels between PjP and PjC groups.

**Figure 3:**
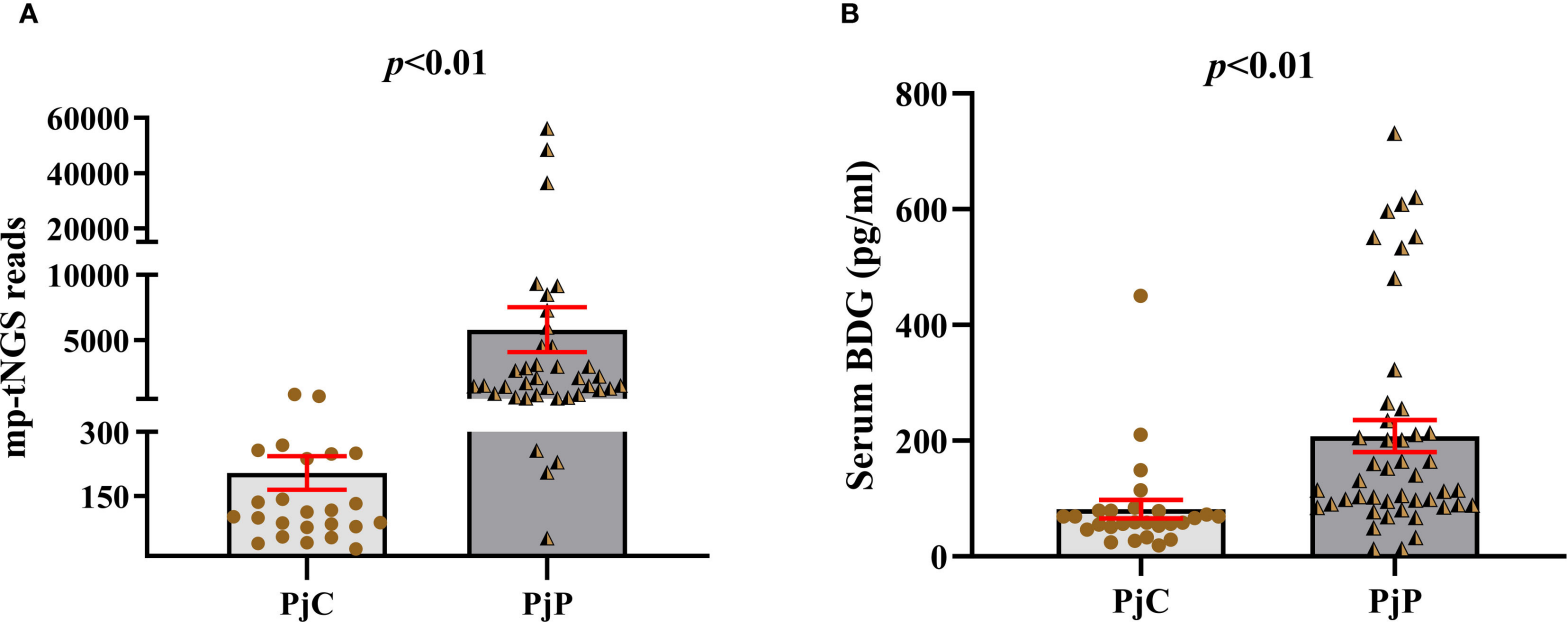
Number of multiplex PCR-based targeted next−generation sequencing (mp-tNGS) reads and levels of serum 1,3-β-D-glucan (BDG) between the *Pneumocystis jirovecii* pneumonia (PjP) and *Pneumocystis jirovecii* colonisation (PjC) groups. **(A)** Number of mp-tNGS reads between the PjP and PjC groups. **(B)** Level of serum BDG between the PjP and PjC groups.

As detailed in **Table 1**, the age was significantly younger in the PjP group, with a mean age of 61.5 ± 10.9 *vs*. 64.2 ± 10.3 in the PjC group (*p* = 0.000) and a higher proportion of male patients in PjP group (*p* = 0.02). No significant differences were noted between the two groups regarding smoking and drinking history (*p* = 0.41, *p* = 0.47). Compared with the PjC group, patients in the PjP group had a significantly higher incidence of fever (54.3% *vs*. 29.6%, *p* = 0.04) and hypoxemia (43.5% *vs*. 18.5%, *p* = 0.03). No significant differences were observed in the incidence of these underlying diseases between the PjP and PjC groups (*p >*0.05); but the proportion of patients with diabetes mellitus was significantly higher in the PjC group compared to the PjP group (25.9% *vs*. 6.5%, *p* = 0.02). Compared with the PjC group, bilateral ground glass opacities on chest CT were more common in the PjP group (50.0% *vs*. 11.1%, *p* = 0.001); typical chest CT findings are shown in **Supplementary Figure A–D**. Compared with the PjC group, patients in the PjP group had significantly lower levels of hemoglobin and albumin (*p* = 0.000). Notably, no significant differences were observed in inflammatory markers, including lymphocyte counts, ESR, LDH and PCT levels, between patients with PjP and those with PjC (**Figures 2A–D**, **Table 1**). In contrast, leukocyte counts, IL-6 and hs-CRP levels were significantly elevated in the PjP group compared to the PjC group (**Figures 2E–G**, **Table 1**). The fungal load was significantly higher in the PjP group, demonstrating a median mp-tNGS read of 1522.00 (581.5, 4898.0), compared to 117.00 (79.00, 257.00) in the PjC group (*p* < 0.0001, **Figure 3A**). Similarly, serum BDG levels were significantly elevated in the PjP group, with a median level of 122.5 (88.75,239.3) pg/ml, versus 59.00 (51.0,79.0) pg/ml in the PjC group (*p <*0.0001, **Figure 3B**).

**Table 1:** Demographics and clinical characteristics of *Pneumocystis jirovecii* pneumonia (PjP) and *Pneumocystis jirovecii* colonisation (PjC) patients (n=73).

Characteristics	Total (n= 73)	PjP (n= 46)	PjC (n= 27)	χ^2^/t/Z	*p-*value
Age (years). mean ± SD	62.5 ± 10.8	61.5 ± 10.9	64.2 ± 10.3	-8.05	0.000
Gender (Male/Female), n	61/12	42/4	19/8	5.43	0.02
Smoking history (≥ 5 years), n (%)	45(61.6)	30(65.2)	15(55.6)	0.67	0.41
Drinking history (≥ 3 years), n (%)	31(42.5)	21(45.7)	10(37.0)	0.52	0.47
Main clinical symptoms, n (%)					
Fever	33(45.2)	25(54.3)	8(29.6)	4.20	0.04
Cough	56(76.7)	37(80.4)	19(70.4)	0.97	0.33
Dyspnea	44(60.3)	29(63.0)	15(55.6)	0.40	0.53
Hypoxemia	25(34.2)	20(43.5)	5(18.5)	4.71	0.03
Underline diseases, n (%)					
AIDS/HIV advanced stage	3(4.1)	3(6.5)	0	1.84	0.18
Diabetes	10(13.7)	3(6.5)	7(25.9)	5.4	0.02
Chronic liver disease	10(13.7)	7(15.2)	3(11.1)	0.24	0.62
Chronic kidney diseases	3(4.1)	2(4.3)	1(3.7)	0.02	0.89
Solid organ malignancy	15(20.5)	11(23.9) ^▴^	4(14.8) ^▴▴^	0.86	0.35
Hematologic tumor	2(2.7)	2(4.3) ^#^	0	1.21	0.27
Autoimmune disease	5(6.8)	3(6.5) ^*^	2(7.4) ^**^	0.02	0.89
Corticosteroid use	4(5.5)	2(4.3)	2(7.4)	0.31	0.58
Chemotherapy	9(12.3)	6(13.0)	3(11.1)	0.06	0.81
Chronic pulmonary disease	18(24.7)	13(28.3) ^△^	5(18.5) ^△△^	0.87	0.35
Radiologic abnormalities, n (%)					
Bilateral ground glass opacities	26(35.6)	23(50.0)	3(11.1)	11.22	0.001
Consolidations	26(35.6)	15(32.6)	11(40.7)	0.49	0.48
Infiltrates	33(45.2)	19(41.3)	14(51.9)	0.76	0.38
Cystic lesions	2(2.7)	2(4.3)	0	1.21	0.27
Pleural effusions	3(4.1)	2(4.3)	1(3.7)	0.02	0.89
Laboratory tests, n (%)					
WBC (×10^9^/L; NR:3.5-9.5)	8.26(5.92,10.62)	8.72 (6.45,10.79)	6.37(4.70,9.40)	-5.75	0.000
Lymphocyte (×10^9^/L; NR: 1.1-3.2)	1.44(1.00,1.97)	1.37(0.99,2.22)	1.60(1.00,1.88)	-3.23	0.747
Hemoglobin (g/L; NR:115-175)	131.70 ± 22.79	130.29 ± 23.93	134.01 ± 20.60	-7.66	0.000
Albumin (g/L; NR:40-55)	35.71 ± 6.00	34.60 ± 5.50	37.45 ± 6.35	-11.98	0.000
ESR (mm/h; NR:0-20)	61.00(28.00,87.0)	61.00(37.00,86.00)	87.00(26.00,103.00)	-0.45	0.653
IL-6 (pg/ml; NR:0-6.6)	420.00(82.49,1965.0)	578.60(100.90,1965.0)	155.80(34.91,352.40)	-25.04	0.000
Hs-CRP (mg/L; NR:0-10)	86.95(55.83,177.30)	93.97(63.81,217.92)	55.46(35.13,100.77)	-17.51	0.000
LDH (U/L; NR:100-240)	261.80(211.90,417.10)	260.50(204.90,440.10)	280.50(216.40,342.00)	-5.06	0.613
PCT (ng/ml; NR: 0-0.5)	2.71(0.49,4.80)	2.82(1.18,3.81)	2.49(0.35,5.03)	-1.77	0.077

In **Table 1**, continuous variables with a normal distribution, expressed as mean ± standard deviation (SD), were compared using the independent samples t-test, with the statistic denoted as *t*. Non-normally distributed continuous variables, expressed as median (interquartile range, 25th–75th percentiles), were compared using the Mann–Whitney U test, with the statistic denoted as *Z*. Categorical variables, expressed as frequencies and percentages [n (%)], were compared using the chi-square test, with the statistic denoted as χ².

AIDS, acquired immune deficiency syndrome; HIV, human immunodeficiency virus; WBC, white blood cell; IL-6, Interleukin-6; ESR, erythrocyte sedimentation rate; Hs-CRP, hypersensitive-c-reactive-protein; LDH, lactate dehydrogenase; PCT, procalcitonin; NR, normal range.

^▴^included one malignant thymoma, one gastric carcinoma, one case of lower esophageal malignancy with secondary mediastinal lymph node metastasis, one case of renal cell carcinoma with secondary bone metastasis, two cases of pulmonary squamous cell carcinoma, one case of breast carcinoma with secondary extramammary metastasis, one case of right temporal lobe malignancy, one case of pulmonary adenocarcinoma, one case of hepatocellular carcinoma, and one case of gastroesophageal junction malignancy;^▴▴^included two hepatocellular carcinomas, one bladder carcinoma, and one pulmonary adenocarcinoma.

^#^ included one case with acute myeloid leukemia (AML) who underwent allogeneic hematopoietic stem cell transplantation (allo-HSCT) and 1 case with angioimmunoblastic T-cell lymphoma (AITL).

^*^ included one patient with connective tissue disease, one patient with rheumatoid arthritis, and one patient with polymyositis. ^**^ included one patient with Sjögren’s syndrome and one patient with systemic lupus erythematosus (SLE).

^△^ included eight patients with interstitial lung disease (ILD), four patients with old pulmonary tuberculosis, and two patients with chronic obstructive pulmonary disease (COPD); ^△△^ included four patients with ILD and one patient with COPD.

### 3.2 Diagnostic efficacy of mp-tNGS and serum BDG for PjP

**Figure 4:**
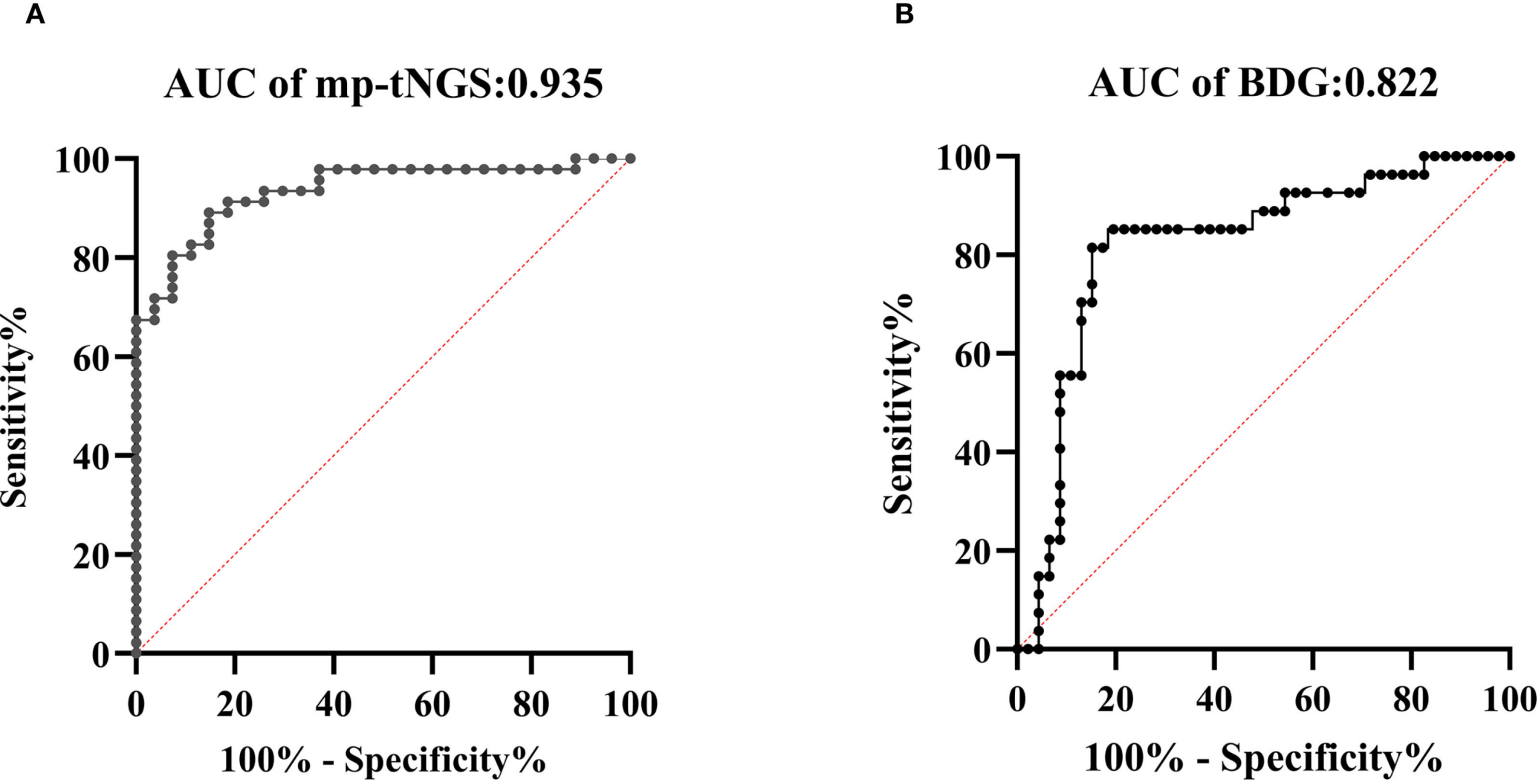
Receiver operating characteristic (ROC) curves were generated to evaluate the diagnostic performance of multiplex PCR-based targeted next−generation sequencing (mp-tNGS) and serum 1,3-β-D-glucan (BDG) assay in differentiating *Pneumocystis jirovecii* pneumonia (PjP) from *Pneumocystis jirovecii* colonization (PjC), (diagonal of ROC is usually referred to as the “no recognition rate line” or the “random guess line”). **(A)** The AUROC for mp-tNGS was 0.935 **(A)**, **(B)** The AUROC for serum BDG assay was 0.822 **(B)**.

The AUROC of BALF mp-tNGS for discriminating PjP from colonization was 0.935 (95% CI: 0.88–0.99), whereas for serum BDG, it was 0.822 (95% CI: 0.72–0.93). The optimal cut-off values for distinguishing *P. jirovecii* infection from colonization were determined to be 355 reads for mp-tNGS (sensitivity: 89.1%; specificity: 85.2%; **Figure 4A**) and 84.5 pg/ml for serum BDG (sensitivity: 85.2%; specificity: 80.4%; **Figure 4B**). These optimal cut-off values were derived from a mathematical assessment of the ROC curves using an optimization analysis of Youden’s J statistic.

### 3.3 The optimal threshold values for differentiating *P. jirovecii* infection from colonization

**Figure 5:**
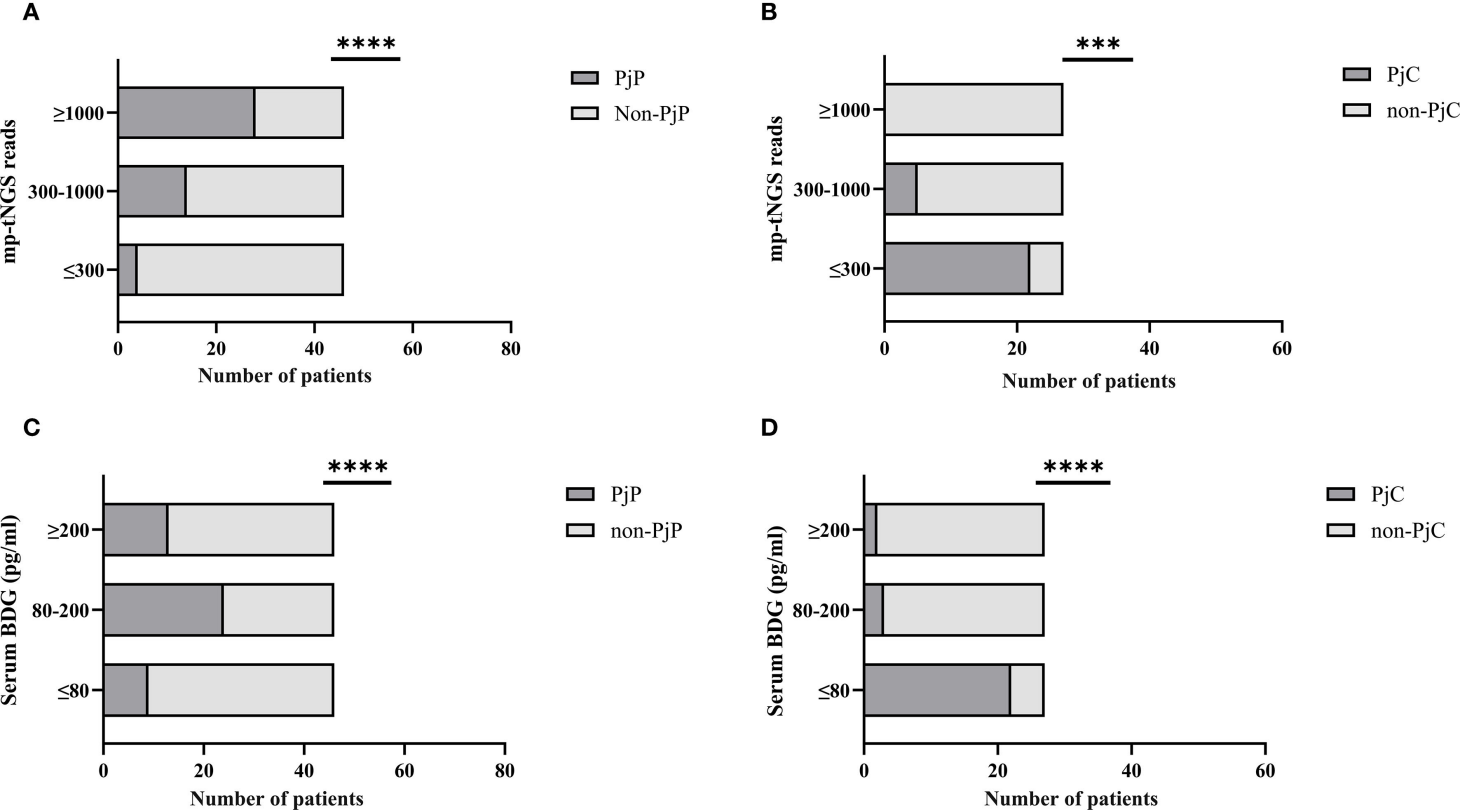
Proportion of *Pneumocystis jirovecii* pneumonia (PjP) and *Pneumocystis jirovecii* colonization (PjC) patients across varying multiplex PCR-based targeted next−generation sequencing (mp-tNGS) read counts and serum 1,3-β-D-glucan (BDG) titers. **(A)** Proportion of PjP cases stratified by mp-tNGS read counts. **(B)** Proportion of PjC cases stratified by mp-tNGS read counts. **(C)** Proportion of PjP cases stratified by serum BDG titers. **(D)** Proportion of PjC cases stratified by serum BDG titers. ***p = 0.003; ****p = 0.000.

**Figure 6:**
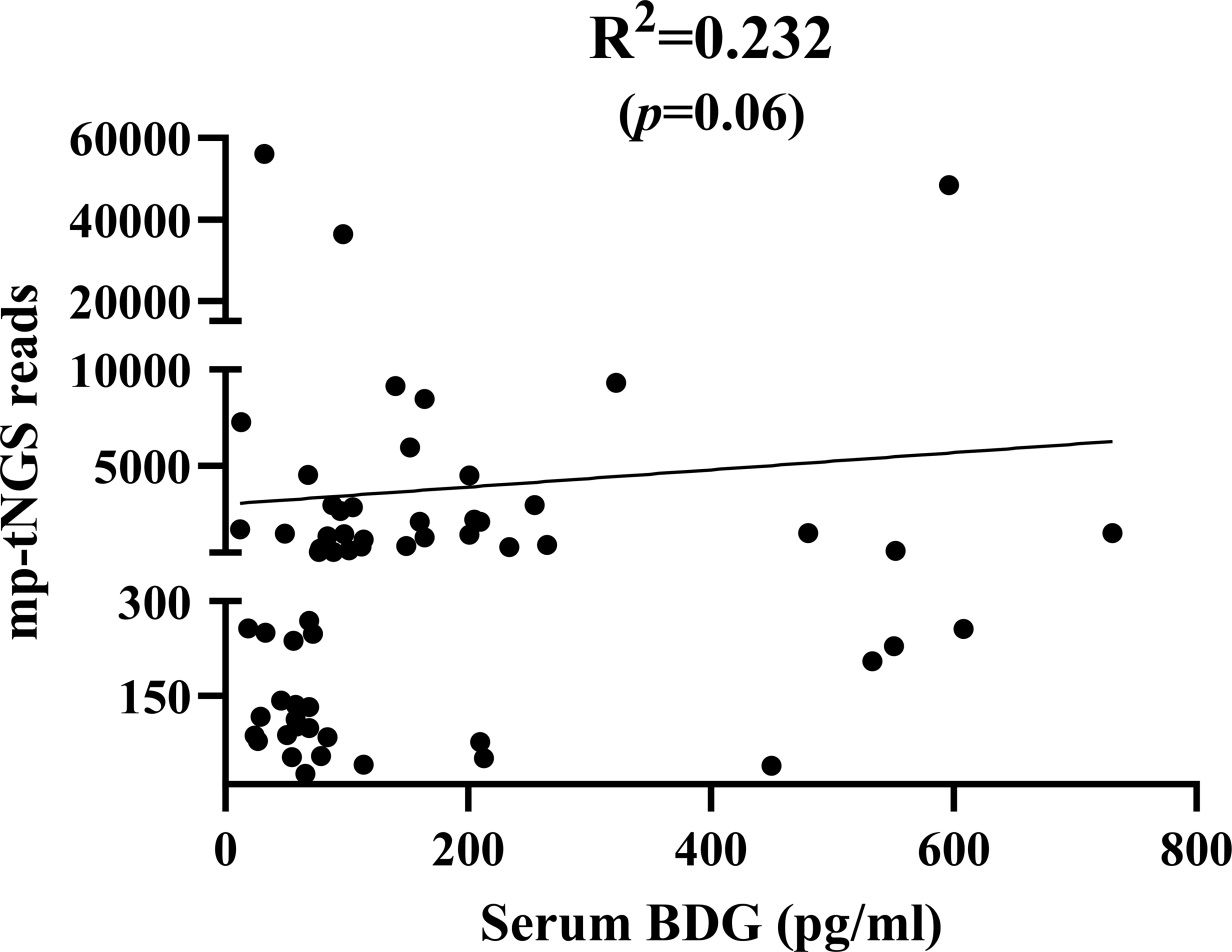
Scatterplot analysis of the correlation between multiplex PCR-based targeted next−generation sequencing (mp-tNGS) read counts and serum 1,3-β-D-glucan (BDG) levels in mp-tNGS-positive patients.

When mp-tNGS read counts were categorized into low (≤300), medium (300–1000), and high (≥1000) ranges, the proportions of patients diagnosed with PjP were 8.7% (4/46), 30.4% (14/46), and 60.9% (28/46), respectively (χ² =28.44, *p* = 0.000; **Figure 5A**). Conversely, among patients with PjC, the proportions were 81.5% (22/27), 18.5% (5/27), and 0% (0/27) in the low, medium, and high read count categories, respectively (χ² =44.33, *p* = 0.000; **Figure 5B**). When serum BDG levels were stratified into low (≤80 pg/ml), medium (80–200 pg/ml), and high (≥200 pg/ml) categories, the proportions of patients with PjP were 19.6% (9/46), 52.2% (24/46), and 28.3% (13/46), respectively (χ² =11.80, *p* = 0.003; **Figure 5C**). In contrast, among patients with PjC, the proportions were 81.5% (22/27), 11.1% (3/27), and 7.4% (2/27) in the low, medium, and high BDG categories, respectively (χ² =42.33, *p* = 0.000; **Figure 5D**). The scatterplot analysis revealed no significant correlation between mp-tNGS read counts and serum BDG levels in mp-tNGS-positive patients (r²=0.232, *p* = 0.06; **Figure 6**).

We evaluated a dual-tests approach for diagnosing PjP using combined thresholds: serum BDG ≥84.5 pg/mL AND mp-tNGS ≥355 reads. This strategy demonstrated 84.2% sensitivity (95% CI: 68.8%-93.9%), correctly identifying PjP cases, and 80.0% specificity (95% CI: 28.4%-99.5%), correctly identifying non-PjP cases. Positive results indicated 97.0% probability of true PjP (PPV; 95% CI: 84.6%-99.5%), while negative results excluded PjP in only 40.0% of cases (NPV; 95% CI: 22.1%-66.1%). Overall diagnostic accuracy ((true positives) + (true negatives))/((true positives + false positives + true negatives + false negatives)) was 83.7% (95% CI: 69.3%-93.2%; **Table 2**). For distinguishing PjC, we tested the inverse criteria: serum BDG <84.5 pg/mL AND mp-tNGS <355 reads. This combination achieved 86.9% sensitivity (95% CI: 66.4%-97.2%) for detecting PjC but only 25.0% specificity (95% CI: 0.6%-80.6%) for excluding non-PjC cases. Positive results indicated 86.9% probability of true PjC (PPV; 95% CI: 78.7%-92.3%), whereas negative results ruled out PjC in merely 25.0% of instances (NPV; 95% CI: 4.3%-71.1%). The overall accuracy was 77.8% (95% CI: 57.7%-91.4%; **Table 3**).

**Table 2:** Performance of combined serum BDG and BALF mp-tNGS in all PjP patients (n=46).

Parameters	PjP mp-tNGS ≥355 reads	PjP mp-tNGS <355 reads
BDG (≥84.5 pg/ml)	32	1
BDG (<84.5 pg/ml)	6	4
Total	38	5
Sensitivity	84.2%	95% CI (68.8%-93.9%)
Specificity	80.0%	95% CI (28.4%-99.5%)
PPV	97.0%	95% CI (84.6%-99.5%)
NPV	40.0%	95% CI (22.1%-66.1%)
Diagnostic accuracy	83.7%	95% CI (69.3%-93.2%)

BDG, (1,3)-β-D-glucan; mp-tNGS, multiplex polymerase chain reaction (PCR)-based targeted next-generation sequencing; PjC, *Pneumocystis jirovecii* pneumonia; PPV, positive predictive value, NPV, negative predictive value, AUROC, area under the receiver operator characteristic curve.

**Table 3:** Performance of combined serum BDG and BALF mp-tNGS in all PjC patients (n=27).

Parameters	PjC mp-tNGS <355 reads	PjC mp-tNGS ≥355 reads
BDG (<84.5 pg/ml)	20	3
BDG (≥84.5 pg/ml)	3	1
Total	23	4
Sensitivity	86.9%	95% CI (66.4%-97.2%)
Specificity	25.0%	95% CI (0.6%-80.6%)
PPV	86.9%	95% CI (78.7%-92.3%)
NPV	25.0%	95% CI (4.3%-71.1%)
Diagnostic accuracy	77.8%	95% CI (57.7%-91.4%)

BDG, (1,3)-β-D-glucan; mp-tNGS, multiplex polymerase chain reaction (PCR)-based targeted next-generation sequencing; PjC, *Pneumocystis jirovecii* colonization; PPV, positive predictive value, NPV, negative predictive value, AUROC, area under the receiver operator characteristic curve.

The clinical and radiological outcomes of patients with PjP showed varying degrees of improvement following treatment with trimethoprim-sulfamethoxazole (TMP-SMX) alone or in combination with other drugs, representative chest CT findings are shown in **Supplementary Figure E–H**.

## 4 Discussion

*Pneumocystis jirovecii* is a fungal pathogen that causes opportunistic pulmonary infections, particularly in immunocompromised individuals (Thomas and Limper, 2004; Grønseth et al., 2021). Notably, PjP is a life-threatening infection in the absence of specific treatment, whereas PjC is a less-severe presentation of *Pneumocystis* infection (Damiani et al., 2013). Therefore, there is an urgent need to develop novel diagnostic approaches to accurately distinguish between *Pneumocystis jirovecii* colonization and active infection. mp-tNGS is a highly sensitive method for detecting *Pneumocystis jirovecii* (Liu et al., 2024; Yin et al., 2024). However, it remains challenging to differentiate between colonization and true infection. Therefore, based on a comprehensive assessment of clinical features, the established cut-off value of mp-tNGS can be utilized to guide the initiation or discontinuation of anti-Pneumocystis jirovecii therapy.

In our study, 36.9% (n=27) of *Pneumocystis jirovecii*-positive patients were identified as colonization. This finding was consistent with previous reports, including a 40% colonization rate observed by Y. Jiang et al (Jiang et al., 2025). and 37.5% (24/64) documented by S. Tasaka et al (Tasaka et al., 2014); but differed from 11.7% (15/128) reported by Matsumura and colleagues (Matsumura et al., 2012), this differ is likely due to the inclusion of 60 pneumonia patients without colonization (non-colonization) in their overall sample, which expanded the sample size and consequently resulted in a lower colonization rate. Several populations have been considered as being colonized by *Pneumocystis jirovecii*, including immunocompromised individuals with varying degrees of immunodeficiency, patients with acute or chronic pulmonary diseases, pregnant women experiencing immunological changes, and healthcare workers frequently exposed to patients with PjP (Morris and Norris, 2012). In our study, the underlying diseases align with the reported risk factors predisposing individuals to colonization.

Various studies have investigated serum BDG as a noninvasive adjunct biomarker for diagnosing PjP in both PLWHand non-HIV-infected individuals, consistently demonstrating an elevated serum BDG levels in individuals with PjP (Tasaka et al., 2007; Cuétara et al., 2008; Pisculli and Sax, 2008; Watanabe et al., 2009; Held et al., 2011). And further studies have demonstrated that patients with active *Pneumocystis jirovecii* infection exhibit significantly higher serum BDG levels compared to those with *P. jirovecii* colonization (Matsumura et al., 2012; Damiani et al., 2013; Tasaka et al., 2014; Liu et al., 2021). Consistent with their observations, our study also found that serum BDG levels were significantly higher in the PjP group compared to the PjC group. Furthermore, we observed that PLWH with PjP had a higher serum BDG level than non-HIV-infected individuals with PjP, which is compatible with the observation by Tasaka and colleagues (Tasaka et al., 2014). This finding may be attributed to the higher fungal burden of *Pneumocystis jirovecii* in the lungs of PLWHwith PjP. Despite the high sensitivity of the BDG assay, false-positive results may occur in the presence of certain confounding factors, including other fungal infections, hemodialysis, Gram-negative bacteremia, severe mucositis, or the administration of intravenous immunoglobulin and the use of certain antibiotics (Onishi et al., 2012; Karageorgopoulos et al., 2013; Del Corpo et al., 2020). However, the high negative predictive value (NPV) of 96% for serum BDG suggests that levels below 80 pg/mL can almost rule out PjP, as previously concluded by Alanio et al (Alanio et al., 2016). Our findings are consistent with those of previous studies and indicate that the BDG assay is useful for excluding PjP. In our study, we established a serum BDG cut-off value of 84.5 pg/mL for distinguishing colonization from active infection; this threshold demonstrated a sensitivity of 85.2% and a specificity of 80.4%. Our findings are consistent with the results reported by Zhang et al (Zhang et al., 2025), who concluded that serum BDG showed high pooled sensitivity (0.83, 95% CI 0.77-0.88) but lower specificity (0.78, 95% CI 0.69-0.85).

To date, only two studies have analyzed the use of mp-tNGS for detecting pathogens in pulmonary infections, highlighting its superior performance in identifying *Pneumocystis jiroveci* (Liu et al., 2024; Yin et al., 2024). As reported by YIN Y et al (Yin et al., 2024). in a prospective cohort study comparing the detection rate of pathogens in 251 BALF samples between tNGS and mNGS, and found that mNGS performed better in detecting rare pathogens (such as *Rhizobium budding*, *Aspergillus Niger* complex, *etc.*), but missed a lot of *P. jirovecii*; while tNGS detected more viruses and *P. jirovecii*. Moreover, they demonstrated that the TAT for mp-tNGS was 10.3 hours, which was significantly shorter than the TAT of 16–24 h for mNGS. Importantly, compared to mp-tNGS, mNGS is limited by higher costs, interference from human genetic material, and the requirement for separate detection of DNA and RNA (Miller et al., 2019). Our results suggest that the combination of the mp-tNGS tests, applying cut-off values of 355 reads, and serum BDG detection, applying an 84.5 pg/ml threshold, can effectively differentiate *Pneumocystis jirovecii* pneumonia from colonization. However, five patients diagnosed with PjP revealed mp-tNGS reads below 355 (51, 205, 229, 256 and 349, respectively). Therefore, when mp-tNGS reads are fewer than 355, the results must be interpreted cautiously in conjunction with clinical manifestations and radiological features. The observed results—specifically, five non-HIV-infected immunocompromised patients with PjP showing mp-tNGS reads below 355 and four PjC case with read above 355—can be attributed to fungal burdens of *P. jirovecii*. As previously reported, the expression of PjP occurs with a lower fungal load in non-HIV-infected immunocompromised patients than PLWH (Limper et al., 1989; Fauchier et al., 2016). In our study, microscopic examination with GMS staining confirmed three cases of PjP, two of which were in PLWH, while none of the colonized or non-colonized patients had HIV infection. Consistent with previous reports, non-HIV-infected patients often develop PjP with a lower fungal load compared to PLWH (Limper et al., 1989), which can lead to false-negative results on microscopic examination (Azoulay et al., 2009).

Previous studies have suggested that serum BDG levels can be used to evaluate fungal load. However, Held et al (Held et al., 2011). demonstrated that BDG levels do not correlate with *Pneumocystis jirovecii* load. Consistent with these findings, our analysis revealed no correlation between mp-tNGS reads and BDG levels in mp-tNGS-positive patients, further supporting the notion that BDG may not reliably reflect *P. jirovecii* burden. A multicenter retrospective study by H. Sun et al (Sun et al., 2022). revealed that BALF-mNGS achieved a sensitivity of 97.40% and specificity of 85.12% for diagnosing PjP, which significantly outperforming blood BDG/LDH and BALF microscopy in sensitivity and BDG/LDH in specificity (*p*<0.05). Despite demonstrating good sensitivity, a notable limitation of their study is the lack of validation in distinguishing between colonization and infection. In this study, when using a combined diagnostic approach with a BDG threshold of 84.5 pg/mL and 355 mp-tNGS reads for the diagnosis of PjP, the sensitivity reached 84.2%, the PPV was 97.0%, and the diagnostic accuracy was 83.7%, which strongly support the diagnosis of PjP. Furthermore, the combination of BDG<84.5 pg/mL and mp-tNGS<355 reads for the diagnosis of PjC achieved a sensitivity of 86.9% and a PPV of 86.9%. These findings demonstrate that the combined use of serum BDG and BALF mp-tNGS provides excellent performance for distinguishing between colonization and infection with *P jirovecii*.

As previously reported, patients with PjP showed a high prevalence of mixed pulmonary infections (Jiang et al., 2021), we hypothesize that this may be attributed to varying degrees of immunosuppression in PjP patients. mp-tNGS has advantages in the identification of multiple microorganisms in respiratory samples, especially in the lower respiratory tract (Yin et al., 2024). In our study, BALF mp-tNGS detected one or more mixed microorganisms in 36 patients with PjP. Among these, cytomegalovirus (CMV) was the most frequently identified, followed by Epstein-Barr virus (EBV), *Streptococcus mitis*, and influenza A virus (H1N1) and herpes simplex virus (HSV). Clinically, we consider these microorganisms less likely to be primary pathogens but rather potential colonizers. In this study, we did not perform a detailed evaluation of these patients, as the primary focus was to differentiate between colonization and infection by *P jirovecii*.

The application of tNGS encounters several substantial challenges, particularly in resource-limited settings. One of the primary barriers is the high upfront investment required for tNGS platforms, reagents, and bioinformatics infrastructure, which can be prohibitively expensive for many laboratories. Additionally, the tNGS workflow is inherently complex, involving multiple steps such as nucleic acid extraction, library preparation, sequencing, and data analysis (Yin et al., 2024); each of these steps introduces potential sources of error or variability, further complicating the process. Moreover, many laboratories, especially those in low- and middle-income countries (LMICs), often lack the necessary infrastructure and resources to effectively implement tNGS, limiting its accessibility and utility in these regions. Our study has several limitations, including its retrospective, single-center design and the relatively small sample size. In the near future, we will provide a detailed report on the other detected mixed microorganisms in PjP.

In conclusion, the combination of BALF mp-tNGS results and serum BDG levels can effectively differentiate between *P jirovecii.* infection and colonization, potentially providing valuable guidance for clinical decision-making and therapeutic strategies.

## Data Availability

The original contributions presented in the study are included in the article/**Supplementary Material**. Further inquiries can be directed to the corresponding authors.

## Supplementary material

Supplementary Figure Representative radiological characteristics of the chest in patients with PjP. Case 1: **(A)** Chest CT of a 62-year-old male revealed diffuse ground-glass opacities in both lungs, with significant improvement observed following treatment with sulfamethoxazole **(E)**. Case 2: **(B)** Chest CT of a 61-year-old male demonstrated symmetrical perihilar high-density opacities and surrounding ground-glass opacities with indistinct margins; after sulfamethoxazole treatment, the lesions nearly resolved **(F)**. Case 3: **(C)** Chest CT of a 44-year-old male showed consolidation in the left lower lobe with an air bronchogram sign, which significantly decreased after sulfamethoxazole therapy **(G)**. Case 4: **(D)** Chest CT of a 61-year-old female revealed patchy opacities, ground-glass opacities, and partial consolidation in both lungs, with significant reduction in lesions following sulfamethoxazole treatment **(H)**.
