# Supplementary figures and images for: Combined multiplex polymerase chain reaction-based targeted next−generation sequencing and serum 1, 3-β-D-glucan for differential diagnosis of Pneumocystis pneumonia and Pneumocystis colonization
Source: Front Cell Infect Microbiol. 2025 Sep 18;15:1611391. doi: 10.3389/fcimb.2025.1611391 (PMC12489942; doi:10.3389/fcimb.2025.1611391)

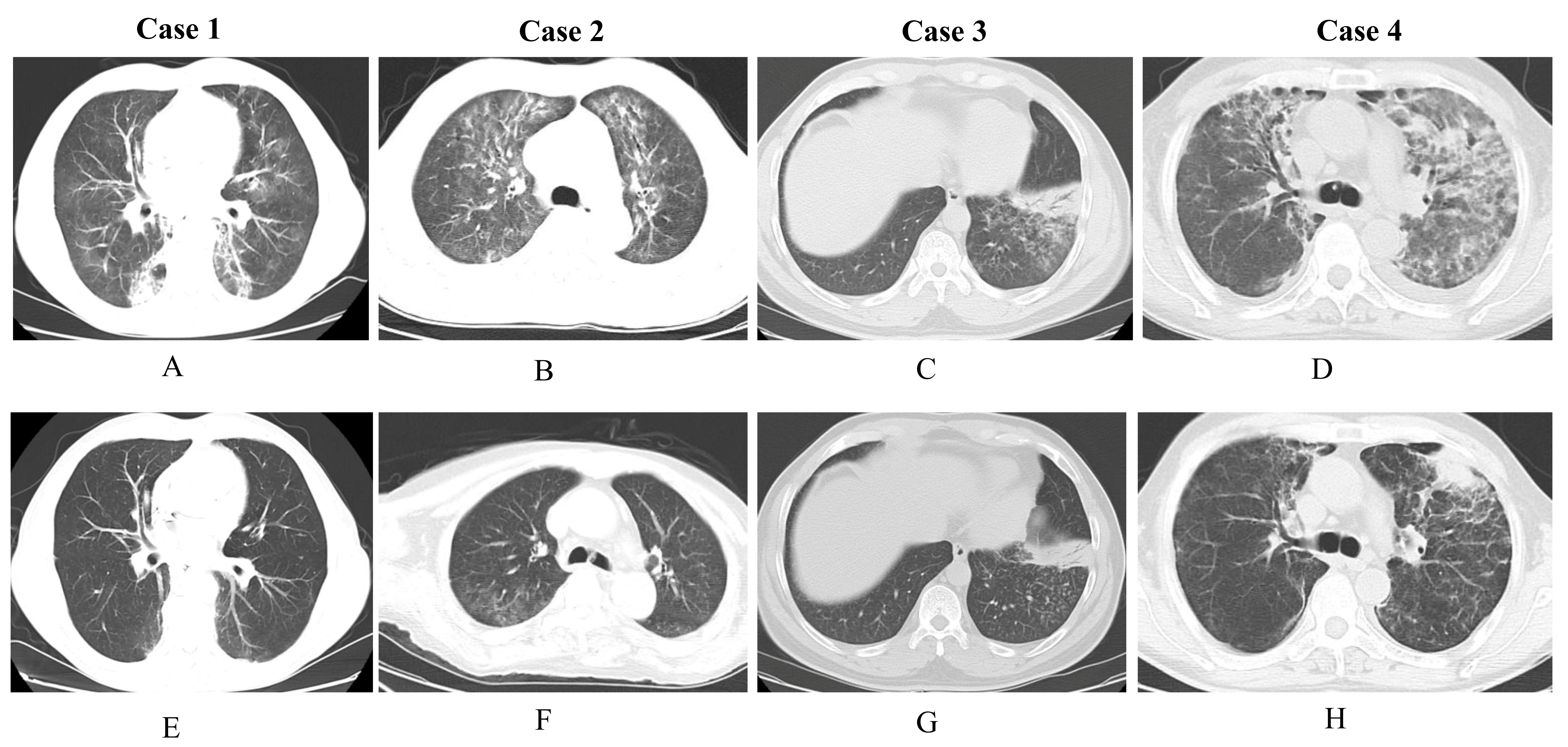

Supplement: Supplementary Figure — Representative radiological characteristics of the chest in patients with PjP. Case 1: (A) Chest CT of a 62-year-old male revealed diffuse ground-glass opacities in both lungs, with significant improvement observed following treatment with sulfamethoxazole (E). Case 2: (B) Chest CT of a 61-year-old male demonstrated symmetrical perihilar high-density opacities and surrounding ground-glass opacities with indistinct margins; after sulfamethoxazole treatment, the lesions nearly resolved (F). Case 3: (C) Chest CT of a 44-year-old male showed consolidation in the left lower lobe with an air bronchogram sign, which significantly decreased after sulfamethoxazole therapy (G). Case 4: (D) Chest CT of a 61-year-old female revealed patchy opacities, ground-glass opacities, and partial consolidation in both lungs, with significant reduction in lesions following sulfamethoxazole treatment (H). [file Image1.tif]
