# Supplementary material for: Combined multiplex polymerase chain reaction-based targeted next−generation sequencing and serum 1, 3-β-D-glucan for differential diagnosis of Pneumocystis pneumonia and Pneumocystis colonization
Source: Front Cell Infect Microbiol. 2025 Sep 18;15:1611391. doi: 10.3389/fcimb.2025.1611391 (PMC12489942; doi:10.3389/fcimb.2025.1611391)
Supplement: Supplementary file 2 [file Table1.docx]

| Targeted Detection of Respiratory Pathogens-List of Pathogenic Microorganism Detection (198 species) | | | |
| --- | --- | --- | --- |
| **Gram-positive bacteria** | Streptococcus pneumoniae | Mycobacterium tuberculosis complex | Corynebacterium diphtheriae |
|  | Streptococcus pyogenes | Staphylococcus aureus | Nocardia * |
|  | Streptococcus agalactis | Whipple's handicap body | Micromonas parvum |
|  | Streptococcus angina * | Cryptobacterium pyogenes | Rhodococcus equi |
|  | Non-tuberculous mycobacteria* |  |  |
| **Gram-negative bacteria** | Klebsiella pneumoniae | Serratia marcescens | Fusobacterium necroticum |
|  | Klebsiella acidogenes | Proteus mirabilis | Fusobacterium nucleatum |
|  | Klebsiella aerogenes | Stenotrophomonas maltophilia | Bacteroides fragilis |
|  | Klebsiella mutans | Haemophilus influenzae | Brucella * |
|  | Pseudomonas aeruginosa | Anopheles Elizabeth | Burkholderia cepacia complex * |
|  | Escherichia coli | Elizabeth aureobacter meningeal sepsis | Burkholderia melioides |
|  | Enterobacter cloacae complex | Moraxella catarrhalis | Burkholderia melioides |
|  | Acinetobacter baumannii | Bordetella pertussis | Legionella * |
|  | Acinetobacter joni | Hombaut's bacteria | Neisseria meningitidis |
|  | Acinetobacter ursin | Bautella parapotussis | Pasteurella multocida |
| **DNA virus** | Herpes simplex virus type 1 (HSV-1) | Human herpesvirus 7 (HHV-7) | Human adenovirus * |
|  | Herpes simplex virus type 2 (HSV-2) | Human boca virus type 1 | Human parvovirus B19 |
|  | Varicella-zoster virus (VZV) | Human boca virus type 2 | JC polyomavirus (JCPyV) |
|  | EB virus (EBV) | Human boca virus type 3 | WU polyomavirus (WUPyV) |
|  | Cytomegalovirus (CMV) | Human boca virus type 4 | BK polyomavirus (BKPyV) |
|  | Human herpesvirus 6 (HHV-6) * |  |  |
| **RNA virus** | Human respiratory syncytial virus type A | Influenza A virus * | Human metapneumovirus |
|  | Human respiratory syncytial virus type B | Influenza B virus * | Rhinovirus * |
|  | Human coronavirus 229E | Influenza C virus | Enterovirus * |
|  | Human coronavirus HKU1 | Human respiratory virus type 1 (human parainfluenza virus type 1) | Measles virus |
|  | Human coronavirus NL63 | Human mumps virus type 2 (human parainfluenza virus type 2) | Rubella virus |
|  | Human coronavirus OC43 | Human respiratory virus type 3 (human parainfluenza virus type 3) | Mumps virus |
|  | Novel coronavirus * | Human mumps virus type 4 (human parainfluenza virus type 4) |  |
| **Fungi** | Candida albicans | Cryptococcus neoformans | **Basilica marneffei** |
|  | Candida tropicalis | Cryptococcus Gert | Mucor racemosa |
|  | Candida pseudosmoothia | Histoplasma capsulatum | Mucor irregularly |
|  | Candida subsmoothie | **Pneumocystis yerschii** | Saiduospora * |
|  | Candida glabra | **Aspergillus fumigatus** | Transverse Aureobasidium * |
|  | Pichia pastoris kudri (Candida koroensis) | **Aspergillus flavus complex** | Rhizopus * |
|  | Pichia pastoris | **Aspergillus Niger complex *** | Rhizomucella * |
|  | Trichosporum Asasi | **Aspergillus terrestrium complex *** | Fusarium * |
| **Mycoplasma, chlamydia**  **Body, Rickettsia, etc** | Mycoplasma pneumoniae | Chlamydia pneumoniae | Bodies Benakox |
|  | Ureaplasma parvosum | Chlamydia trachomatis | Chlamydia psittaci |
|  | Ureaplasma urealyticum |  |  |
| Note: * Marker means that the pathogenic microorganism covers one or more types (species, subtype, serotype, etc.). If the test result of the pathogenic microorganism is positive, one of the types or itself is positive, and the specific contents are as follows: | | | |
| Non-tuberculous mycobacteria *: Mycobacterium avium complex, Mycobacterium avium, Mycobacterium intracellularum, Mycobacterium turtle-abscess complex, Mycobacterium turtle, Mycobacterium abscess, Mycobacterium asiatica, Mycobacterium subtle, Mycobacterium Gordon, Mycobacterium kansas, Mycobacterium malmosa, Mycobacterium scrofula, Mycobacterium schneideri, Mycobacterium simi, Mycobacterium sulka, Mycobacterium toad  Bacteria, Mycobacterium fortuitae, Mycobacterium smegmatis. | | | |
| Nocardia *: Nocardia melioides, Nocardia St. George, Nocardia Brazil, Nocardia abscess, Nocardia astrosus, Nocardia sag, Nocardia guinea pig otitis, Nocardia terpene, Nocardia Africa, Nocardia neoformans and other 15 species of Nocardia. | | | |
| Streptococcus angina *: Covering Streptococcus intermedia and other three streptococci. | | | |
| Brucella *: Brucella covered abortion, Brucella canis, Brucella accidentalis and Brucella cetacean. | | | |
| Burkholderia cepacia complex *: 12 species of Burkholderia cepacia, such as covering Burkholderia cepacia, new Burkholderia cepacia, contaminated Burkholderia cepacia and burkholderia polyphaga. | | | |
| Legionella *: Covers 10 kinds of Legionella such as Legionella pneumophila, Legionella bozmann, Legionella Long Beach and Legionella Mickedade. | | | |
| Enterobacter cloacae complex *: covering 5 kinds of Enterobacter such as Enterobacter albescens. | | | |
| Human herpesvirus type 6 (HHV-6) *: Human herpesvirus type 6A (HHV-6A), human herpesvirus type 6B (HHV-6B). | | | |
| Human adenovirus *: It covers 31 types of human adenovirus group B, human adenovirus type 3, human adenovirus type 7, human adenovirus type 11, human adenovirus type 14, human adenovirus type 21, human adenovirus type 34, human adenovirus type 35, human adenovirus type 55, human adenovirus group C, human adenovirus type 1, human adenovirus type 2, human adenovirus type 5, human adenovirus type 6, human adenovirus type 57, human adenovirus group D and human adenovirus type 4. | | | |
| Enterovirus *: It covers 104 types of enterovirus group A, enterovirus group B, enterovirus group C, enterovirus group D, enterovirus A71, enterovirus D68, coxsackievirus A2, coxsackievirus A5, coxsackievirus A6, coxsackievirus A10, coxsackievirus A16, coxsackievirus B3, ECO virus E18 and ECO virus E30. | | | |
| Rhinovirus: rhinovirus type A, rhinovirus type B and rhinovirus type C. | | | |
| Influenza A virus *: Influenza A virus H1N1, Influenza A virus H3N2, Influenza A virus H1N1 (2009) Influenza A virus H5N1, Influenza A virus H7N9. | | | |
| Influenza B virus *: influenza B virus Victoria strain, influenza B virus Yamagata strain. | | | |
| Novel coronavirus *: Covering 23 subdivisions, Novel coronavirus (Alpha_B.1.1.7), novel coronavirus (Beta_B.1.351), novel coronavirus (Delta_B.1.617.2), novel coronavirus (Gamma_P.1), novel coronavirus (Lamda_C.37), novel coronavirus (Mu_B.1.621), novel coronavirus (Omicron_BA.1.1.529), novel coronavirus (Omicron_BA. 2.12.1), novel coronavirus (Omicron_BA.2.75), novel coronavirus (Omicron_BA.5.11), novel coronavirus (Omicron_BA.5.2) Coronavirus (Omicron_XBB.1.5). | | | |
| Saiduospora *: Saiduospora covering the tip and Saiduospora bovi. | | | |
| Horizontal Aureobasidium *: Horizontal Aureobasidium umbrella, Horizontal Aureobasidium | | | |
| Rhizopus *: covering 4 kinds of Rhizopus, such as Rhizopus de, Rhizopus microsporus and Rhizopus oryzae. | | | |
| Rhizomucella *: Rhizomucella minimus. | | | |
| Fusarium *: 8 kinds of Fusarium, such as Fusarium substratum and Fusarium sclerensis. | | | |
| Aspergillus Niger Complex *: Covering Aspergillus Niger. | | | |
| Aspergillus terreus complex *: Aspergillus terreus covered. | | | |
